# Effects of Cynaroside on Cell Proliferation, Apoptosis, Migration and Invasion though the MET/AKT/mTOR Axis in Gastric Cancer

**DOI:** 10.3390/ijms222212125

**Published:** 2021-11-09

**Authors:** Juanli Ji, Zhongze Wang, Wei Sun, Zekun Li, Huarui Cai, Erhu Zhao, Hongjuan Cui

**Affiliations:** 1 State Key Laboratory of Silkworm Genome Biology, Medical Research Institute, Southwest University, Chongqing 400715, China; juanli123@email.swu.edu.cn (J.J.); asd463110343@email.swu.edu.cn (Z.W.); sw1170040034@email.swu.edu.cn (W.S.); a1982365456@email.swu.edu.cn (Z.L.); caihuarui123@email.swu.edu.cn (H.C.); ehzhao@swu.edu.cn (E.Z.); 2 Chongqing Engineering and Technology Research Center for Silk Biomaterials and Regenerative Medicine, Chongqing 400716, China; 3 Engineering Research Center for Cancer Biomedical and Translational Medicine, Southwest University, Chongqing 400715, China; 4 College of Sericulture, Textile and Biomass Sciences, Southwest University, Chongqing 400716, China

**Keywords:** gastric cancer, cynaroside, cell proliferation, migration and invasion, MET

## Abstract

The Chinese medicine monomer cynaroside (Cy) is a flavonoid glycoside compound that widely exists in plants and has a variety of pharmacological effects, such as its important role in the respiratory system, cardiovascular system and central nervous system. Studies have reported that Cy has varying degrees of anticancer activity in non-small cell lung cancer, cervical cancer, liver cancer, esophageal cancer and other cancers. However, there are no relevant reports about its role in gastric cancer. The MET/AKT/mTOR signaling pathway plays important roles in regulating various biological processes, including cell proliferation, apoptosis, autophagy, invasion and tumorigenesis. In this study, we confirmed that Cy can inhibit the cell growth, migration and invasion and tumorigenesis in gastric cancer. Our finding shows that Cy can block the MET/AKT/mTOR axis by decreasing the phosphorylation level of AKT, mTOR and P70S6K. Therefore, the MET/AKT/mTOR axis may be an important target for Cy. In summary, Cy has anti-cancer properties and is expected to be a potential drug for the treatment of gastric cancer.

## 1. Introduction

Gastric cancer is a malignant tumor of the digestive tract originating from the epithelium of the gastric mucosa. In 2020, there will be more than one million new cases and an estimated 769,000 deaths. Among cancers, the incidence of gastric cancer ranks fifth, and the mortality rate ranks fourth [1,2]. Early gastric cancer has no significant symptoms. Due to the lack of effective early diagnosis molecules, most patients are already in the advanced stage at the time of diagnosis [3]. Systemic chemotherapy is the main treatment for patients with gastric cancer. Although chemotherapy prolongs the survival time of patients, the prognosis is still not optimistic. The five-year survival rate of patients with advanced gastric cancer is less than 10% [4]. Therefore, it is urgent to develop highly effective gastric cancer chemotherapy drugs and find effective diagnostic molecules to improve the prognosis of patients.

Cynaroside (Cy), also called luteoloside or luteolin 7-glucoside, is a flavonoid glycoside compound widely found in plants, and mainly found in honeysuckle, snow chrysanthemum, brocade lantern, celery and other plants. The IUPAC name of Cy is 2-(3,4-dihydroxyphenyl)-5-hydroxy-7-[(2*S*,3*R*,4*S*,5*S*,6*R*)-3,4,5-trihydroxy-6-(hydroxymethyl)oxan-2-yl]oxychromen-4-one, and its chemical structure was shown in Figure S1. Cy has a variety of pharmacological properties, such as anti-inflammatory and anti-bacterial properties, scavenging free radicals, anti-oxidation and anti-tumor properties [5,6,7,8,9,10]. Studies have reported that Cy has varying degrees of anti-cancer activity in non-small cell lung cancer, cervical cancer, liver cancer, and esophageal cancer [10,11,12]. However, there is no relevant report about its role in gastric cancer. The receptor tyrosine kinase family plays an important role in cell proliferation, survival, differentiation, migration, transcription and cell cycle regulation [13,14]. Mesenchymal–epithelial transition protein is a member of the RTK family and is an important oncogenic determinant that can regulate the occurrence, development and deterioration of tumors [15]. Similarly, the MET/AKT/mTOR pathway is pivotal in cancer cells and plays an important effect on cell proliferation, apoptosis, autophagy, invasion and tumorigenesis [16,17,18,19,20,21,22]. In this study, we confirmed that Cy can inhibit the proliferation, migration and invasion of gastric cancer cells and induce apoptosis. This effect may be achieved by promoting the ubiquitination and degradation of MET and reducing the protein level of MET. Therefore, Cy may be a potential MET targeted drug for gastric cancer.

## 2. Materials and Methods

### 2.1. Cell Culture

The three gastric cancer cell lines (HGC27, MKN45 and SGC7901) were obtained from Roswell Park Memorial Institute-1640 (RPMI-1640, Gibco, Grand Island, NY, USA), containing 10% fetal bovine serum (FBS; Gibco, Auckland, New Zealand) and 1% penicillin-streptomycin (P/S; Invitrogen, Carlsbad, CA, USA). The 293FT cells were maintained in DMEM supplemented with 1% G418 (Invitrogen, Carlsbad, CA, USA), 2% glutamine (Invitrogen, Carlsbad, CA, USA), 1% non-essential amino acids (Invitrogen, Carlsbad, CA, USA), and 1% sodium pyruvate (Invitrogen, Carlsbad, CA, USA). All cells were incubated in a humidified atmosphere with 5% CO_2_ at 37 °C.

### 2.2. Drug Treatment

Cynaroside (molecular formula: C_21_H_20_O_11_, relative molecular mass: 448.4, purity ≥ 98%) was purchased from Chengdu Mansite Bio-technology Company (Chengdu, Sichuan province, China), and Cy was dissolved in dimethyl sulfoxide (DMSO) as a 200 mM stock solution. Cy was used to treat gastric cancer cells at different concentrations (0, 25, 50, 75 and 100 μM). Microscopy (CKX41SF, Olympus, Tokyo, Japan) was used to detect cell morphology. Simultaneously, a hemocytometer was used for cell number counting. Each experiment was repeated three times.

### 2.3. Cell Viability Assay

Cell viability was tested by MTT (Sigma Aldrich, St. Louis, MI, USA). The log phase cells were seeded into a 96-well plate (1000 cells per well), pre-cultured for 24 h, and treated with different concentrations (0, 25, 50, 75 and 100 µM) of Cy. The control group was treated with DMSO. At different time points, MTT (5 mg/mL, 20 µL per well) was added to the cells and incubate them in a cell culture incubator for 2 h. Then, DMSO (200 µL) was used to dissolve formazan. A microplate reader (Thermo Fisher, Waltham, MA, USA) was used to measure the absorbance of the cells at 560 nm. To calculate cell death rate: Inhibition Rate (%) = (average A560 of the control group − average A560 of the experimental group)/(average A560 of the control group − average A560 of the blank group) × 100%.

### 2.4. BrdU Staining

BrdU staining was used to detect cell proliferation. Cells in logarithmic phase were collected, and 2 × 10^4^ HGC27, MKN45 and SGC7901 gastric cancer cells were seeded into 24-well cell culture plates and placed into a cell incubator overnight. Cy (50 μM) was added to the above cells, and the control group was treated with DMSO. After incubation for 48 h, 10 g/mL BrdU solution (Sigma Aldrich, St. Louis, MO, USA) was added to each well for 2 h. After fixation with 4% paraformaldehyde at room temperature for 20 min, 2 M HCl was added for denaturation, the cells were perforated with 0.3% Triton X-100, and then 10% goat serum was prepared for sealing (ZSGB-Bio, Beijing, China). Next, the primary antibody (Abcam, Cambridge, MA, USA) and the secondary antibody (Abcam) were used for incubation. After incubation, DAPI was added to stain the nuclei. Finally, the fluorescence signal was observed under a fluorescence microscope and photographed. The positive rate of BrdU was calculated by statistical analysis and calculation.

### 2.5. Plate Clone Formation Experiment

The plate colony formation assay was used to detect cell proliferation. Cells in the logarithmic phase were collected, and HGC27, MKN45 and SGC7901 gastric cancer cells were inoculated into a 6-well cell culture plate at 1000 cells per well and placed in a cell culture incubator overnight. Cy (50 µM) was added to the above cells, and the control group was treated with DMSO. After culturing for 2 weeks, the cells were stained with crystal violet for 20 min, placed on a digital scanner for scanning, the results were saved, and statistical analysis was performed.

### 2.6. Flow Cytometry

Flow cytometry was used to detect the cell cycle and apoptosis. Cells were cultured in an incubator under normal conditions, and gastric cancer cells treated with DMSO and Cy at a specific time were collected. To detect the cell cycle, cells were fixed overnight at 4 °C with 75% ethanol. The cells were washed with PBS to remove residual ethanol and incubated with propidium iodide (PI, BD, San Jose, CA, USA) and RNase A (Sigma Aldrich, St Louis, MO, USA) at 37 °C for 1 h. To detect apoptosis, the collected cells were washed with PBS. The cells were then resuspended in 100 μL binding buffer (BD, San Jose, CA, USA). The cells were then incubated with FITC-labeled Annexin V (BD, San Jose, CA, USA) and PI (BD, San Jose, CA, USA) at room temperature for 15 min. All samples were analyzed using FACS C6 (BD, San Jose, CA, USA) with Cell Quest software. Each experiment was repeated three times.

### 2.7. Western Blot Analysis

Western blotting was used to detect the protein expression level. RIPA lysis buffer (Beyotime, Shanghai, China) containing benzene methanesulfonyl fluoride (Beyotime, Shanghai, China) was used to lyse the cells. The cell lysate was then denatured at 100 °C for 30 min. A BCA protein determination kit (Beyotime, Shanghai, China) was used to determine the protein concentration, and 30 mg of protein was subjected to 10% SDS-PAGE to separate the protein. The separated proteins were transferred to PVDF membranes (Millipore, Kenilworth, NJ, USA) and blocked with 5% bovine serum albumin (BSA) at room temperature. Primary antibodies against CDK1, CDK2, Cyclin E1, N-Cadherin, Vimentin, Slug, C-PARP, C-Caspase-3, p-mTOR, p-P70S6K, p-AKT, and MET were purchased from Cell Signaling Technology (Proteintech, Chicago, IL, USA), and tubulin was purchased from Beyotime, and incubated with membranes overnight at 4 °C. Then, the blots were washed an incubated with HRP-conjugated secondary antibodies (goat anti-mouse IgG and goat anti-rabbit IgG, 1:10000, Beyotime, China) at room temperature for 1.5 h. Finally, an ECL system (Beyotime, Shanghai, China) and detection analysis system (Clinx Science, Shanghai, China) were used to visualize and capture proteins.

### 2.8. Transfection and Infection

The 293FT cells were used for transfection. The packaging plasmid (pLP1, pLP2, pLP/VSVG) and the overexpression plasmid pCDH-CMV-MCS-EF1cop GFP-MET (vector encoding human MET was obtained from YouBio, Changsha, China) by Lipofectamine 2000 (Invitrogen, Carlsbad, CA, USA) were co-transfected into 293FT cells. After 48 h of transfection, the virus supernatant was collected and infected with gastric cancer cells with the help of polybrene. Puromycin (2 mg/mL) was used to select successfully transfected cells. Lentiviral production, infection, and establishment of stable human gastric cancer cell lines with the overexpression of the MET gene were performed as previously described [23,24].

### 2.9. The qRT-PCR Assay

After treatment with DMSO or Cy at 37 °C for 48 h, the cells were collected. Total RNA was isolated from the cells using TRIzol reagent (Invitrogen, Carlsbad, CA, USA) according to the manufacturer’s instructions. M-MLV reverse transcriptase (Promega, Madison, WI, USA) was used for reverse transcription. Quantitative real-time fluorescent quantitative PCR (qRT-PCR) was used to detect the relative expression of the MET gene.

### 2.10. Ubiquitination Assay

The 293FT cells treated with MG132 and Cy were collected and lysed using IP lysis buffer antibodies, incubated overnight with lysate and then incubated with IgG (rabbit) to pull down Met proteins. After five washes with PBS, the proteins were denatured at 100 °C for 30 min and then separated using an 8% SDS-PAGE gel. Ubiquitylation of MET was examined using ubiquitination antibodies.

### 2.11. Soft Agar Assay

The soft agar method was used to detect the tumor-forming ability of gastric cancer cells in vitro, which was operated as previously described [25]. The base agar (1.5 mL per well) consisted of 2 × RPMI-1640 (Gibco) and 0.6% agarose (Sigma Aldrich, USA), which was added to a 6-well plate. Then, the RPMI-1640 medium containing 1000 cells, 0.3% agar and Cy was added to the base agar. The cells were incubated at 37 °C for 2–3 weeks, colonies were captured under a microscope, stained with MTT and counted.

### 2.12. Tumor Xenografts

Female NOD/SCID immunodeficient mice approximately 20 days old were purchased from Beijing Weitong Lihua Laboratory Animal Technology Co., Ltd. (Beijing, China) and the mice were kept in the SPF animal room IVC system for approximately a week to help the mice adapt to the environment. The gastric cancer cell lines SGC7901 and MKN45 were injected subcutaneously into mice, and 1 million cells were injected at each point. After 1 week, the mice were randomly divided into two groups. One group was injected with Cy (50 mg/kg) every two days, and the other group was injected with MDSO as a control. At the same time, the volume of the mouse was measured, and the calculation formula was π/6 × length × width^2^. Finally, the tumor was removed, weighed and photographed. All animal experiments were conducted according to the Declaration of Helsinki and were pre-approved and supervised by the Institutional Animal Care and Use Committees of the Southwest University.

### 2.13. Statistical Analysis

All experiments were repeated at least three times and the data are presented as the mean ± SD unless noted otherwise. Differences between data groups were evaluated for significance using Student’s *t*-test of unpaired data or one-way analysis of variance and Bonferroni post hoc test. *p* values less than 0.05 indicate statistical significance.

## 3. Results and Discussion

### 3.1. Cynaroside Inhibits the Proliferation of Gastric Cancer Cells

**Figure 1:**
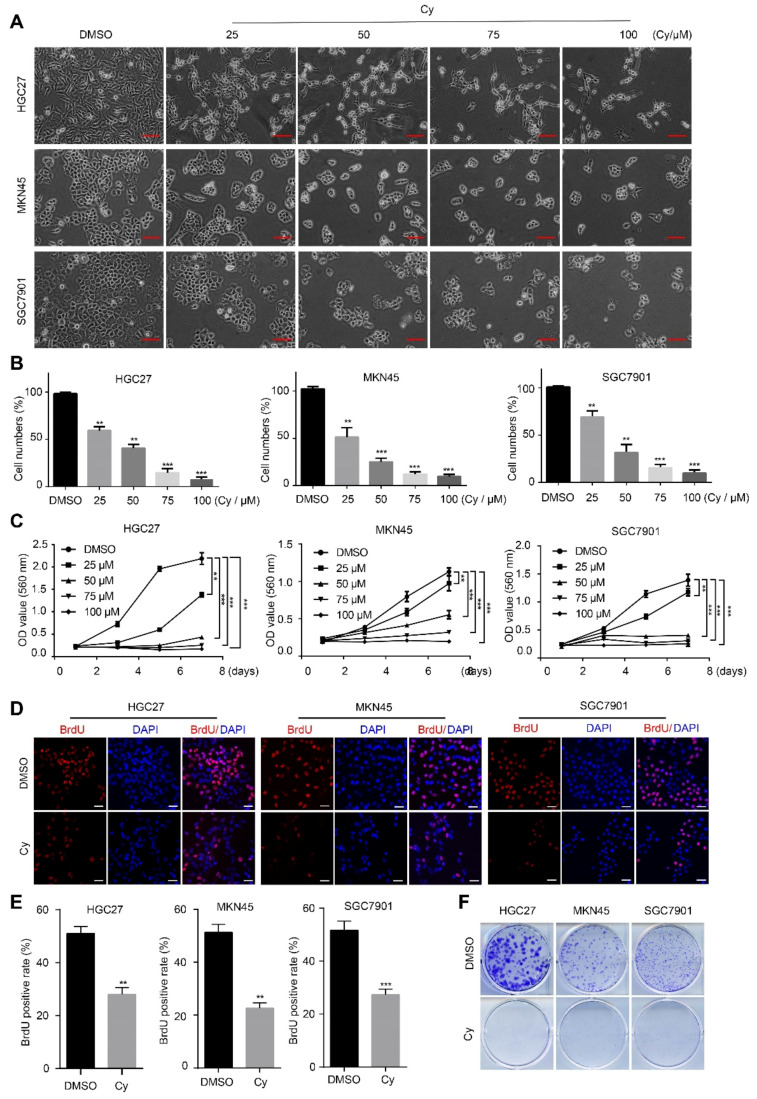
Cynaroside inhibits the growth of gastric cells. (**A**) Phenotypes of HGC27, MKN45 and SGC7901 cells treated with different concentrations of Cy for 48 h. DMSO was added as a control, and the scale was 100 μm. (**B**) The percentage of cells in each group, with the percentage of cells in the control group being 100%. (**C**) HGC27, MKN45 and SGC7901 cells were treated with different concentrations of Cy, and the cell growth was measured by MTT method. (**D**) HGC27, MKN45 and SGC7901 cells were treated with 50 μM Cy for 48 h, with BrdU positive cells, And taking DMSO as a control, scale bar = 100 μm. (**E**) Quantification of BrdU-positive HGC27, MKN45 and SGC7901 cells in (**D**). DMSO was used as a control. (**F**) HGC27, MKN45 and SGC7901 cells were treated with 50 μM Cy for the analysis of colony formation experiments, and DMSO was added as a control. ** *p* < 0.01, *** *p* < 0.001, *p*-values < 0.05 were considered as statistically significant.

Previous studies showed that cynaroside has no significant cytotoxicity on normal cells [26,27,28], so it is a safe and natural compound. In fact, Cy has a variety of pharmacological properties, such as anti-tumor properties. However, there are few reports about its role in gastric cancer. To explore the effect of Cy on the proliferation of gastric cancer cells, we set up different concentration gradient Cy concentrations by consulting the literature, which were 25, 50, 75, and 100 μM. The control group was DMSO. Three gastric cancer cell lines were treated with Cy for 48 h, and then observed under a microscope. The results showed that after treatment with Cy, the quantity of gastric cancer cells changed significantly, and with increasing Cy concentration, the number of cells decreased in a concentration-dependent manner (Figure 1A,B). To verify this result, we performed MTT cell viability assay experiments, BrdU incorporation experiments and plate clone formation experiments. MTT cell viability assay experiments showed that compared with the control group, the growth of the cells in the Cy-added group was significantly inhibited, and with the increase in the Cy concentration, the inhibition of cell growth was more obvious (Figure 1C). The results of the BrdU incorporation experiments showed that compared with the control group, the BrdU positive percentage of the cells in the treatment group was significantly reduced (Figure 1D,E). Plate cloning experiments also showed that the cloning ability of gastric cancer cells was significantly weakened after treatment with Cy (Figure 1F and Figure S2). These results prove that Cy significantly inhibits the growth and proliferation of gastric cancer cells.

### 3.2. Cynaroside Causes Cell Cycle Arrest at S Phase

**Figure 2:**
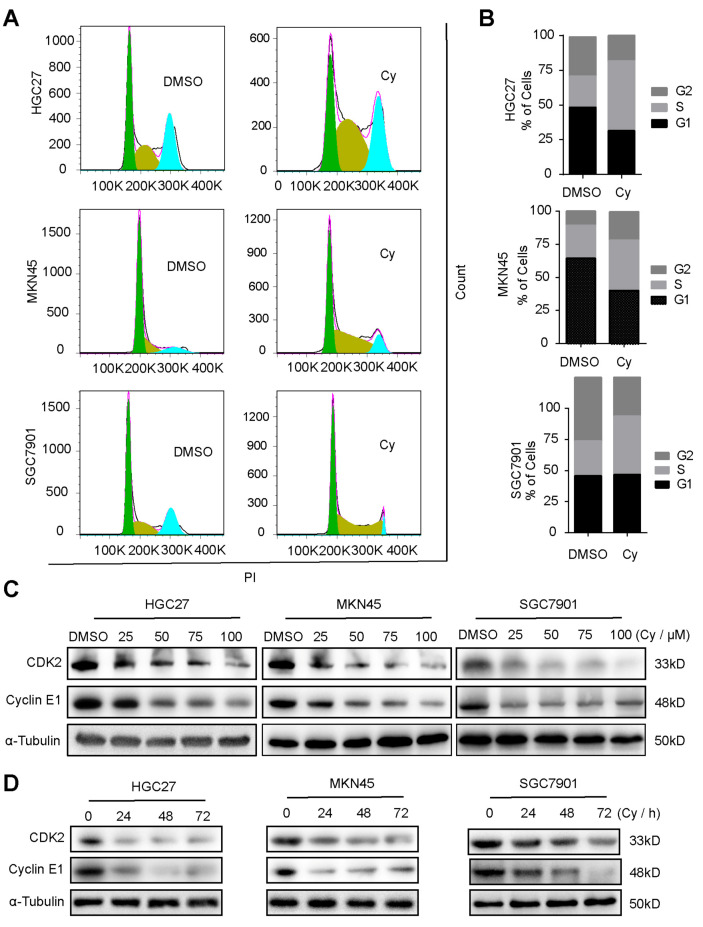
Cynaroside causes cell cycle arrest at S phase. (**A**) After Cy treatment of gastric cancer cells for 48 h, the cell cycle was detected by flow cytometry, and DMSO was used as a control. (**B**) The percentage of results of each cell cycle was statistically drawn about (**A**). (**C**) Cyclin E2 and CDK2 protein expression levels of cells treated with different concentrations of Cy for 48 h; α-Tubulin was used as control. (**D**) The expression of CDK2 and CyclinE2 in gastric cancer cells exposed to 50 μM Cy for different times: 0, 24, 48 and 72 h. α-Tubulin was used as a control.

We further explored the influence of Cy on the cycle progression of gastric cancer cells. We used flow cytometry to detect the changes in the cycle progression of gastric cancer cells after Cy treatment for 48 h. After Cy treatment of the cells, the cycle of gastric cancer cells was obviously blocked in S phase (Figure 2A,B). To verify the inhibitory effect of Cy on the cycle progression of gastric cancer cells, Western blot experiments were performed to detect the changes in the expression of S phase-related cyclin in gastric cancer cells. The experimental results showed that Cy significantly inhibited the expression of CDK2 and CyclinE1, and this inhibition was concentration- (Figure 2C and Figure S3A) and time-dependent (Figure 2D and Figure S3B). These results indicate that Cy induces cycle arrest of gastric cancer cells in S phase.

### 3.3. Cynaroside Inhibits the Migration and Invasion of Gastric Cancer Cells

**Figure 3:**
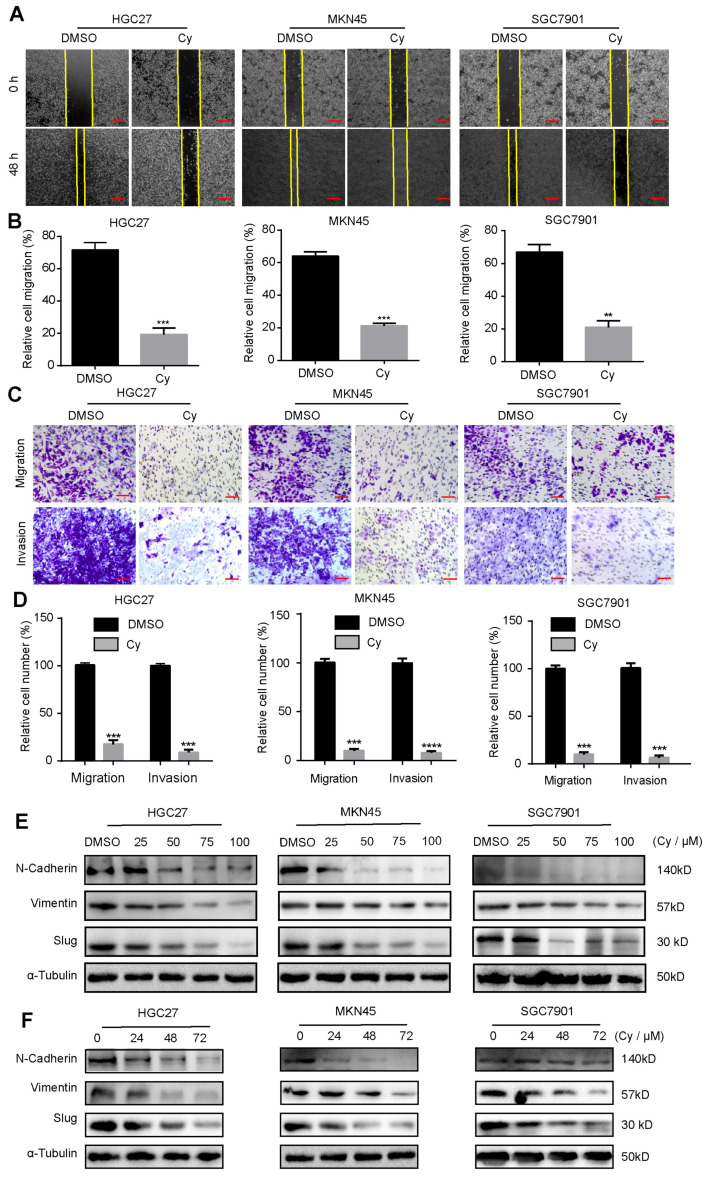
Cynaroside inhibits the migration and invasion of gastric cancer cells. (**A**) The migration ability of Cy-treated gastric cancer cells measured by a wound healing test, with DMSO as a control. (**B**) Calculation of the percentage of cell healing, based on the results in (**A**). (**C**) The Transwell experiment detects the invasion ability of Cy-treated gastric cancer cells, and DMSO is used as a control. (**D**) Calculation of the percentage of cell invasion based on the results in (**C**). (**E**) The protein expression levels of *N*-cadherin, vimentin and slug in cells treated with different concentrations of Cy for 48 h; α-Tubulin was used as control. (**F**) The expression of *N*-cadherin, vimentin and slug in gastric cancer cells exposed to 50 μM Cy for different times: 0, 24, 48 and 72 h. α-Tubulin was used as acontrol. ** *p* < 0.01, *** *p* < 0.001, **** *p* < 0.0001, *p*-values < 0.05 were considered as statistically significant.

Gastric cancer is a common and highly aggressive malignant tumor worldwide. Therefore, we explored the influence of Cy on the migration and invasion of gastric cancer cells. First, we designed a scratch healing experiment to preliminarily detect whether Cy has the ability to inhibit the migration of gastric cancer cells in vitro. The experimental results showed that compared with the control group, the migration ability of gastric cancer cells was significantly weakened after Cy treatment for 48 h (Figure 3A,B). Next, we conducted Transwell experiments to verify the migration and invasion ability of gastric cancer cells with and without Matrigel. The experimental results showed that the migration and invasion ability of gastric cancer cells after Cy treatment for 48 h was significantly lower than that of the control group (Figure 3C,D). Finally, we detected the expression of migration and invasion-related proteins in gastric cancer cells by Western blot. Western blot experiment results are consistent with the above phenomenon experiment. After Cy treatment, the expression of important proteins related to gastric cancer cell migration and invasion was down-regulated, and this down-regulation was concentration-dependent (Figure 3E and Figure S4A) and time-dependent (Figure 3F and Figure S4B).

### 3.4. Cynaroside Induces Apoptosis of Gastric Cancer Cells

**Figure 4:**
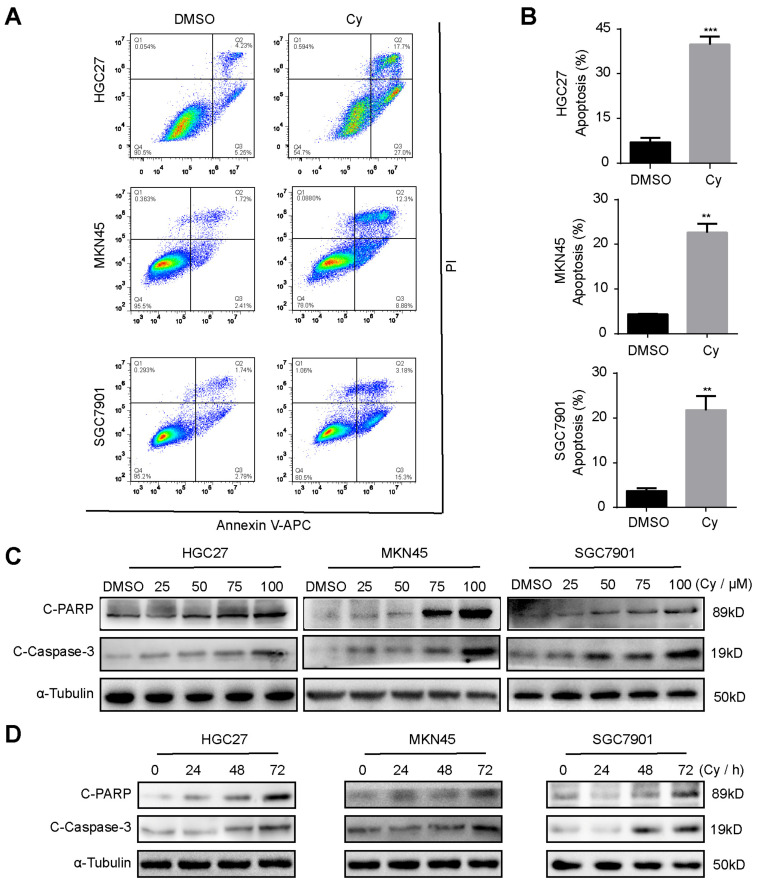
Cynaroside induces apoptosis of gastric cancer cells. (**A**) Flow cytometry was used to detect apoptosis of gastric cancer cells 48 h after Cy treatment, and DMSO was used as a control. (**B**) The percentage of apoptosis rate. (**C**) The protein expression levels of C-PARP and C-caspase-3 in cells treated with different concentrations of Cy for 48 h, α-tubulin was used as control. (**D**) The expression of C-PARP and C-caspase-3 in gastric cancer cells exposed to 50 μM Cy for different times: 0, 24, 48 and 72 h. α-Tubulin was used as a control. ** *p* < 0.01, *** *p* < 0.001, *p*-values < 0.05 were considered as statistically significant.

To test whether Cy can induce apoptosis of gastric cancer cells, we performed flow cytometry experiments. After 48 h of treatment of gastric cancer cells with 50 μM Cy, they were stained with PI and annexin V-APC. The results showed that Cy can significantly induce cell apoptosis (Figure 4A,B). To verify this result, we tested the expression of spliceosomes of apoptosis substrates PARP and caspase-3 by Western blot analysis. The results showed that Cy can up-regulate the protein expression levels of the spliceosome of PARP and caspase-3, and this up-regulation is mainly concentration-dependent (Figure 4C and Figure S5A) and time-dependent (Figure 4D and Figure S5B). These findings are consistent with a previous study which reported that Cy induced the apoptosis through the p53/Bcl-2 pathway in cervical cancer cells [12]. However, Cy can also protect the normal cells against the apoptosis induced by oxidative stress [28,29]. To sum up, the specific activity of inducing apoptosis is one of anti-cancer properties of Cy.

### 3.5. Cynaroside Inhibits the Activation of the AKT/mTOR Pathway by Enhancing MET Ubiquitination Degradation

**Figure 5:**
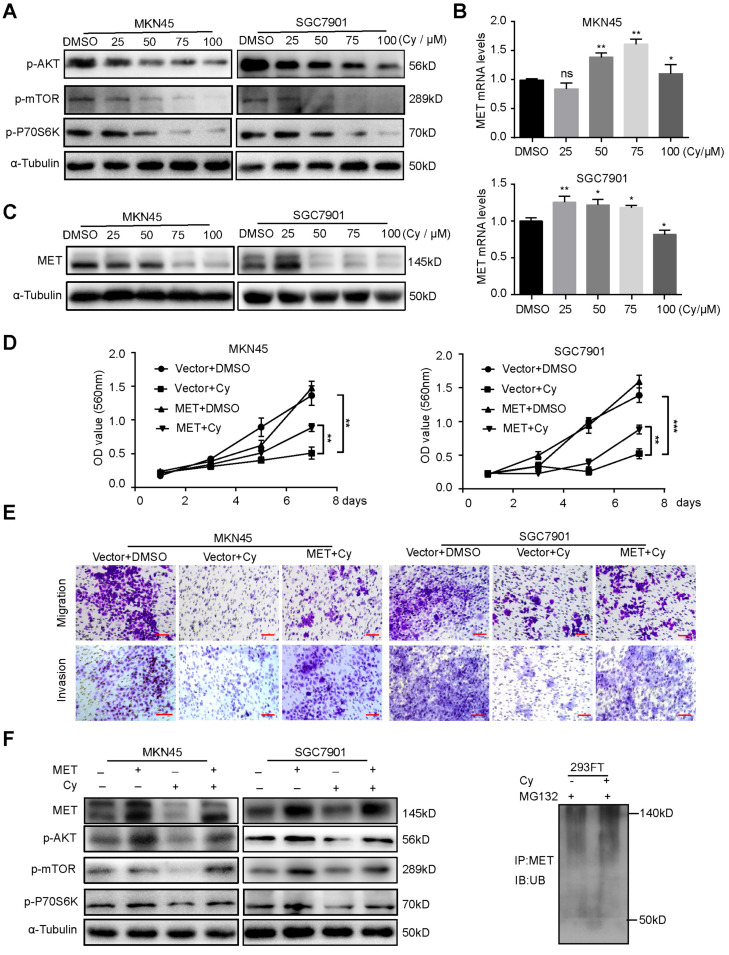
Cynaroside inhibits the activation of the p-mTOR, p-P70S6K and p-AKT pathway by enhancing MET ubiquitination degradation. (**A**) The expression levels of p-mTOR, p-P70S6K and P-AKT proteins in MKN45 and SGC7901 cells were measured by Western blot treated with different concentrations of Cy. α-Tubulin was used as a control. (**B**) After treating the cells with different concentrations of Cy, fluorescence quantitative PCR detects the mRNA level of MET. (**C**) The expression of MET in gastric cancer cells exposed to 50 μM Cy in 48 h. α-Tubulin was used as control. (**D**) Viability of MKN45 and SGC7901 overexpressing MET and vector under treatment with Cy (50 µM), respectively. DMSO was added as a control. (**E**) The migration and invasion of MKN45 and SGC7901 overexpressing MET or empty vector under treatment with Cy (50 µM), respectively. DMSO was added as a control. (**F**) The expression levels of p-mTOR, p-P70S6K and p-AKT proteins in MKN45 and SGC7901 cells which overexpressed MET and vector were measured by Western blot treated with different concentrations of Cy. α-Tubulin was used as control. (G) After 293FT cells were treated with 50 µM Cy, IP analysis was used to detect the ubiquitination level of MET protein. * *p* < 0.05, ** *p* < 0.01, *** *p* < 0.001, *p*-values < 0.05 were considered as statistically significant.

To further study the mechanism by which Cy inhibits the proliferation and migration of gastric cancer cells, we used Western blotting to detect important signaling pathways in gastric cancer cells. The results showed that Cy reduced the phosphorylation levels of AKT, mTOR and p-P70S6K (Figure 5A). The receptor tyrosine kinase (RTK) family was discovered in the 1980s. It is encoded by 58 genes and is divided into 20 subfamilies [30]. They play an important role in cell proliferation, survival, differentiation, migration, transcription, and cell cycle regulation. Mesenchymal–epithelial transition protein (MET) is a member of the RTK family and is an important oncogenic determinant that can regulate the occurrence, development and deterioration of tumors [31]. Therefore, we detected the expression of MET in gastric cancer cells by qRT-PCR and Western blot. The results showed that Cy did not reduce the mRNA expression of MET (Figure 5B), but significantly down-regulated the expression of MET in gastric cancer cells (Figure 5C). As an important tyrosine receptor kinase, MET is reduced at its level and can significantly inhibit the activity of its downstream related pathways. Therefore, we overexpressed MET in MKN45 and SGC7901 cells (Figure S6A) and then treated them with Cy. The above-mentioned cell lines overexpressing MET significantly reduced the inhibition of Cy on cell proliferation (Figure 5D), cell migration and invasion (Figure 5E). In addition, the protein expression levels of AKT, mTOR and p-P70S6K also showed obvious responses (Figure 5F). Generally, there are two ways to down-regulate the protein level: regulation of transcription level, and regulation of protein level (regulation of protein level mainly refers to ubiquitination degradation). We also found that Cy can inhibit the expression level of MET in gastric cancer. First, we detected the mRNA level of MET in gastric cancer cells by fluorescence quantitative PCR. Interestingly, after Cy treatment, the RNA level of MET did not decrease, but in fact increased to a certain extent (Figure 5B). Therefore, we speculated that Cy down-regulated the expression level of the MET protein through the ubiquitination degradation pathway. We conducted an IP experiment for verification, and the results were consistent with expectations. Cy can enhance the ubiquitination level of MET (Figure 5F), and thus, Cy inhibits gastric cancer cell activation of the p-mTOR, p-P70S6K and p-AKT pathways by enhancing MET ubiquitination degradation.

### 3.6. Cynaroside Inhibits the Tumorigenesis Ability of Gastric Cancer Cells In Vivo and In Vitro

**Figure 6:**
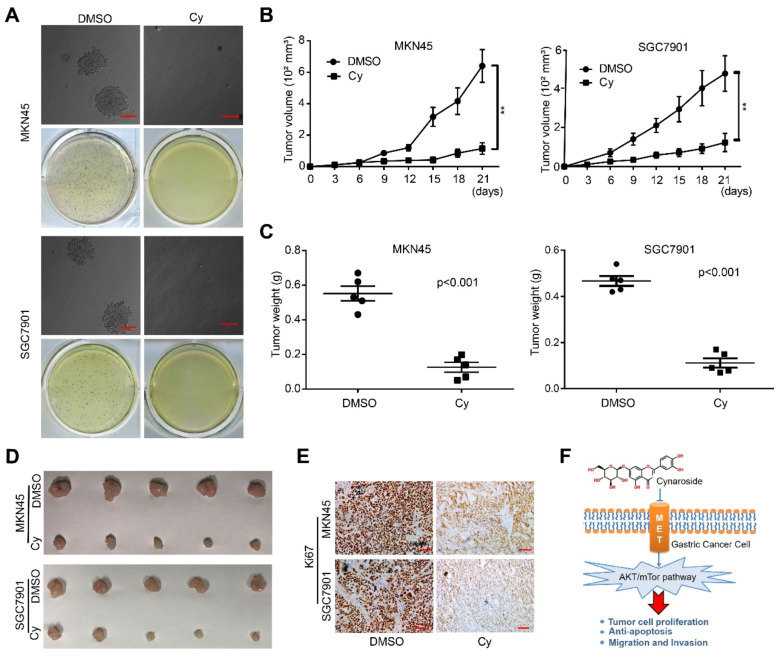
Cynaroside inhibits the tumorigenesis ability of gastric cancer cells in vivo and in vitro. (**A**) The gastric cancer cells were treated with 50 μM Cy, and the soft agar test was used for detection; DMSO was used as a control. (**B**) The volume of the tumor removed from the mice. (**C**) The weight of the tumor removed from the mice. (**D**) Image of mouse xenograft tumor. (**E**) The immunohistochemistry experiment was used to detect the expression level of Ki-67 in mouse tumor tissues. (**F**) Model for effects of Cy in gastric cancer. ** *p* < 0.01, *p*-values < 0.05 were considered as statistically significant.

To verify the effect of Cy on the self-renewal of gastric cancer cells, we tested the tumor-forming ability of gastric cancer cells in vitro through soft agar formation experiments. The results showed that compared with the control group, the number of colonies formed by gastric cancer cells after Cy treatment was significantly reduced. It was found by comparing the clone size. The clones after Cy treatment were significantly smaller than those of the control group (Figure 6A and Figure S7A). Next, we established a subcutaneous xenograft tumor model in NOD/SCID mice and explored the effect of Cy on the tumorigenesis ability of gastric cancer to fully explain the anti-cancer activity of Cy. Compared with the control group, the body weights of the mice did not change significantly (Figure S7B). However, the volumes of the tumors in the administration group were significantly smaller than those in the control group (Figure 6B), and the tumor weights were also significantly reduced (Figure 6C,D). IHC staining showed that the expression of the cell proliferation marker Ki67 in the Cy treatment group was significantly reduced (Figure 6E). These results indicate that Cy significantly inhibits the tumorigenic ability of gastric cancer cells. In summary, Cy has anti-tumor activity effects on gastric cancer cells (Figure 6F).

## 4. Conclusions

Gastric cancer is a common malignant tumor of the digestive system [32]. Risk factors for this disease include *Helicobacter pylori* infection, age, high salt intake, and insufficient fruit and vegetable diet [33,34]. Although advanced diagnosis and treatment methods have reduced its incidence in some developed countries, gastric cancer is still the main cause of cancer deaths in East Asia (including China, Japan and Korea) [35]. Therefore, it is urgent to strengthen the research and development of chemotherapeutic drugs. Traditional Chinese medicine originated in China, and an increasing number of studies have shown that traditional Chinese medicine plays an important role in adjuvant treatment of gastric cancer, alleviating patients’ symptoms, and improving survival rates.

Cy is a flavonoid glycoside compound widely found in plants [6]. This study explored the role of Cy in the development of gastric cancer cells and its possible regulatory mechanism. MET is an important oncogenic determinant that can regulate the occurrence, development and deterioration of tumors [15]. Similarly, the MET/AKT/mTOR signaling pathway plays important roles in regulating various biological processes, including cell proliferation, apoptosis, autophagy, invasion and tumorigenesis [16,17,19,20,21,22]. Our findings showed that Cy can decrease the protein levels of p-mTOR, p-P70S6K and p-AKT. Moreover, we found that Cy can significantly reduce gastric cancer cell self-renewal ability and inhibit its tumorigenesis ability in vivo. Therefore, the MET/AKT/mTOR axis may be an important target for Cy to inhibit the growth and migration of gastric cancer cells. Furthermore, the AKT/mTOR targeted therapy has become a hotspot of anti-tumor therapy, with a variety of drugs such as AKT or mTOR kinase inhibitors in clinical development of gastric cancer. Cy can also block the activation of the MET/AKT/mTOR axis, so it may be a potential therapeutic agent in the clinical treatment of gastric cancer.

In summary, Cy has shown great anti-cancer activity in gastric cancer in vivo and in vitro, which demonstrates Cy as a clinically targeted drug for gastric cancer patients, and will provide a preliminary theoretical basis for the treatment of gastric cancer. However, there are still problems that are worthy of future research, such as how Cy induces apoptosis in gastric cancer cells and the specific mode of action of Cy on MET downregulation. We still need to conduct in-depth studies to clarify the specific mechanism that Cy plays a role in gastric cancer.

## Data Availability

The data presented in this study are available on request from the corresponding author.

## Supplementary Materials

The following are available online at https://www.mdpi.com/article/10.3390/ijms222212125/s1.

## Abbreviations

AKT	serine/threonine kinase 1
C-PARP	cleaved poly (ADP-ribose) polymerase 1
CCNE	cyclin E
CDKs	cyclin-dependent kinases
Cy	cynaroside
DAPI	4′,6-diamidino-2-phenylindole
DMEM	Dulbecco’s modified Eagle’s medium
DMSO	dimethyl sulfoxide
FBS	fetal bovine serum
IgG	immunoglobulin G
IHC	immunohistochemistry
IUPAC	International Union of Pure and Applied Chemistry
MET	mesenchymal–epithelial transition protein
mTOR	mammalian target of rapamycin
MTT	3-(4,5-dimethylthiazol-2-yl)-2,5-diphenyl tetrazolium bromide
NOD/SCID	nonobese diabetic/severe combined immunodeficiency
PI	propidium iodide
PVDF	polyvinylidene difluoride
RTK	receptor tyrosine kinase
SPF	specific pathogen-free
