# Supplementary material for: Effects of Cynaroside on Cell Proliferation, Apoptosis, Migration and Invasion though the MET/AKT/mTOR Axis in Gastric Cancer
Source: Int J Mol Sci. 2021 Nov 9;22(22):12125. doi: 10.3390/ijms222212125 (PMC8618935; doi:10.3390/ijms222212125)
Supplement: Supplementary file 1 [file ijms-22-12125-s001.zip › ijms-1405373-supplementary.pdf]

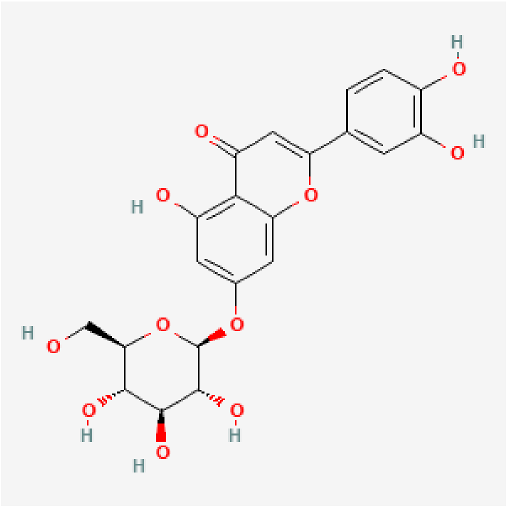

Figure S1 Structures of Cynaroside (PubChem Compound Database).

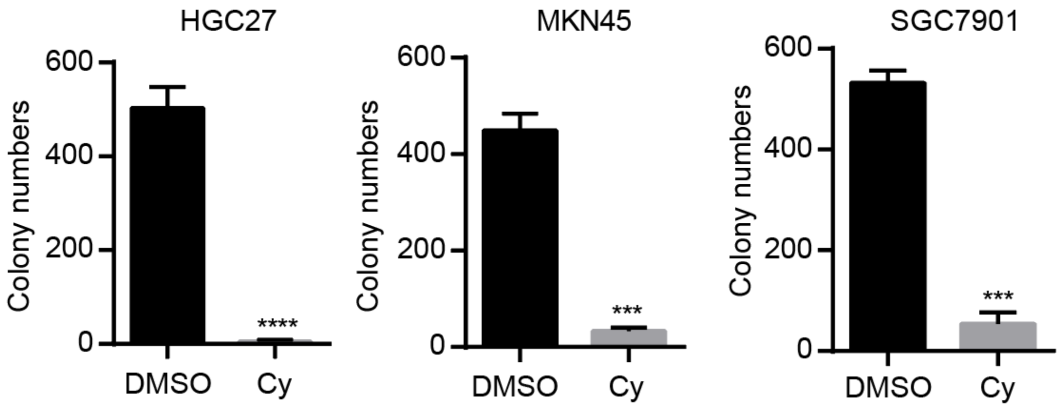

Figure S2 Quantification of the number of HGC27, MKN45 and SGC7901 cell clones in Figure 1f

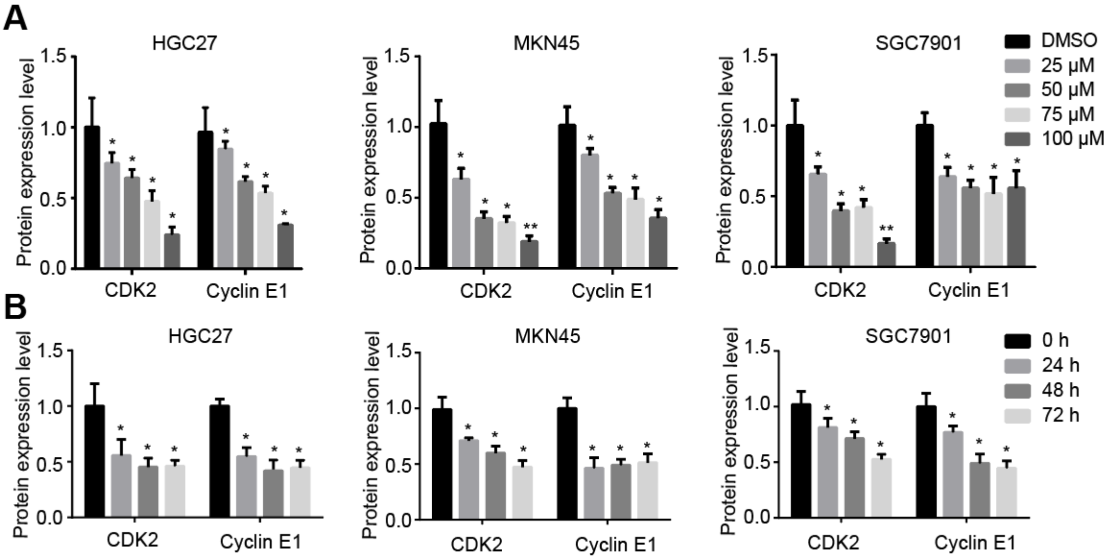

Figure S3 (A) Quantification of CDK2 and CyclinE2 protein expression of cells treated with different concentrations of Cy for 48 h in Figure 2C; (B) Quantification of CDK2 and CyclinE2 protein expression of cells exposing to 50 μM Cy in different time for 0, 24, 48 and 72 h in Figure 2D.

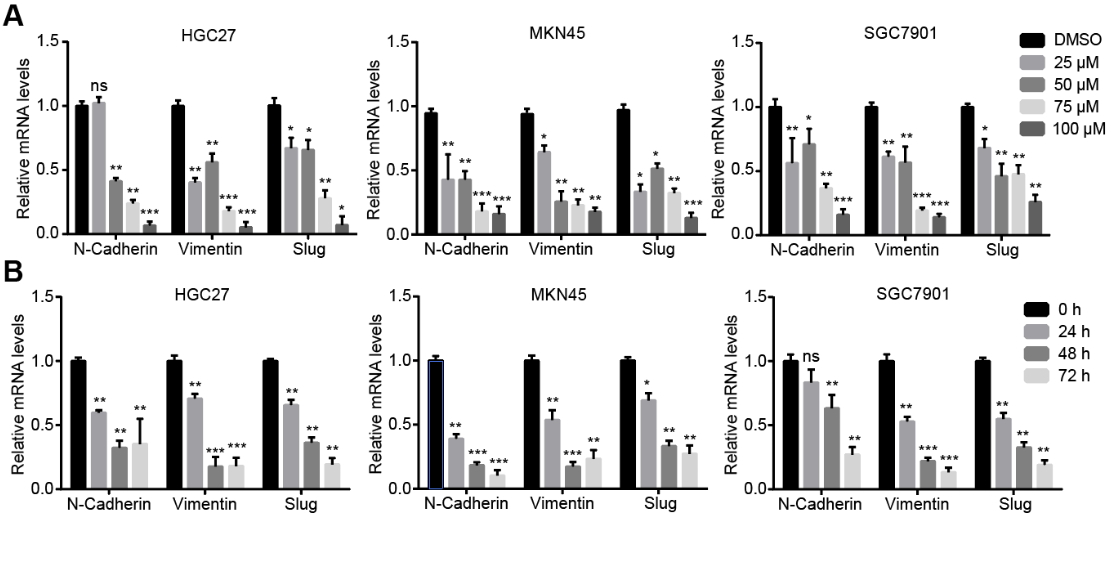

Figure S4 (A) Quantification of N-Cadherin, Vimentin and Slug protein expression of cells treated with different concentrations of Cy for 48 h in Figure 3E; (B) Quantification of N-Cadherin, Vimentin and Slug protein expression of cells exposing to 50 μM Cy in different time for 0, 24, 48 and 72 h in Figure 3F.

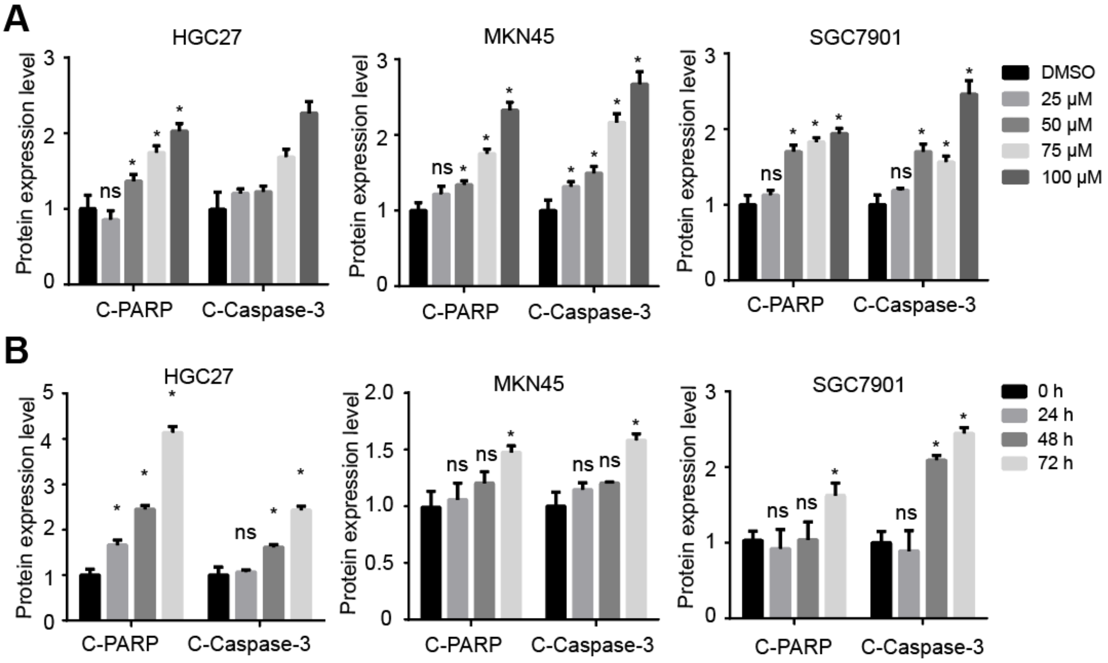

Figure S5 (A) Quantification of C-PARP and C-Caspase-3 protein expression of cells treated with different concentrations of Cy for 48 h in Figure 4C; (B) Quantification of C-PARP and C-Caspase-3 protein expression of cells exposing to 50 μM Cy in different time for 0, 24, 48 and 72 h in Figure 4D.

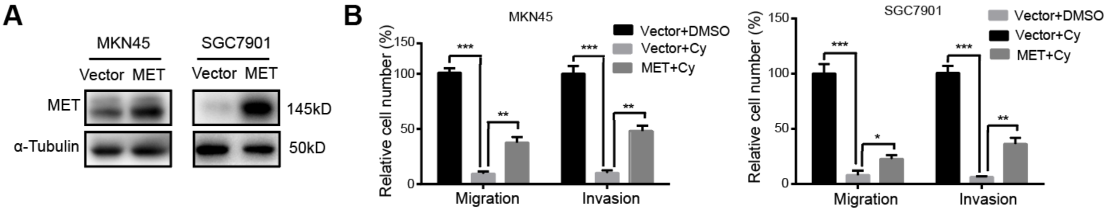

Figure S6 (A) The expression of MET in MKN45 and SGC7901 overexpressing MET and vector under treatment with Cy (50 μM), respectively. DMSO was added as a control. (B) Quantification of migration and invasion in MKN45 and SGC7901 overexpressing MET and vector under treatment with Cy (50 μM), respectively. DMSO was added as a control.

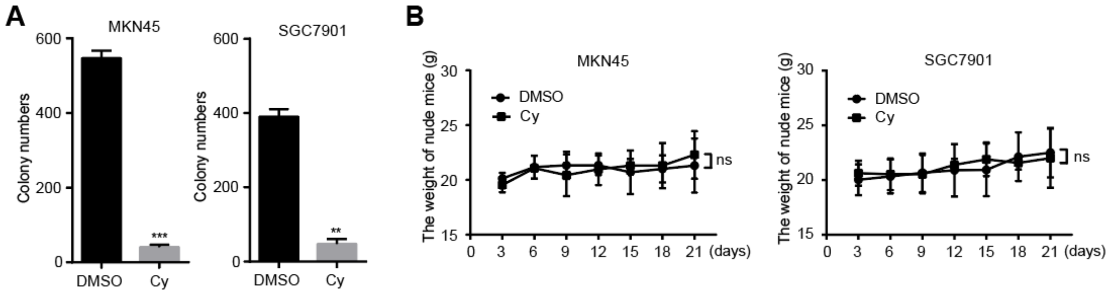

Figure S7 (A) Quantification of colonies in soft agar test in Figure 6A. (B) The weight of the nude mice.
